# Deciphering STAT3’s negative regulation of LHPP in ESCC progression through single-cell transcriptomics analysis

**DOI:** 10.1186/s10020-024-00962-0

**Published:** 2024-10-28

**Authors:** Yurao Chen, Zemao Zheng, Luoshai Wang, Ronghuai Chen, Ming He, Xiang Zhao, Liyan Jin, Juan Yao

**Affiliations:** 1 Department of Radiation Oncology, Huaian Hospital of Huaian City, Huaian, 223299 Jiangsu China; 2 Department of Radiation Oncology, Huaian Cancer Hospital, Huaian, 223299 Jiangsu China; 3 Department of Thoracic Surgery, Huaian Hospital of Huaian City, Huaian, 223299 Jiangsu China; 4 Department of Oncology, Wujin Hospital Affiliated with Jiangsu University, Changzhou, 213000 Jiangsu China; 5 Department of Oncology, The Wujin Clinical college of Xuzhou Medical University, Changzhou, 213000 Jiangsu China

**Keywords:** ESCC, Single-cell transcriptomics, Tumor microenvironment, STAT3, LHPP

## Abstract

**Background:**

Esophageal Squamous Cell Carcinoma (ESCC) remains a predominant health concern in the world, characterized by high prevalence and mortality rates. Advances in single-cell transcriptomics have revolutionized cancer research by enabling a precise dissection of cellular and molecular diversity within tumors.

**Objective:**

This study aims to elucidate the cellular dynamics and molecular mechanisms in ESCC, focusing on the transcriptional influence of STAT3 (Signal Transducer and Activator of Transcription 3) and its interaction with LHPP, thereby uncovering potential therapeutic targets.

**Methods:**

Single-cell RNA sequencing was employed to analyze 44,206 cells from tumor and adjacent normal tissues of ESCC patients, identifying distinct cell types and their transcriptional shifts. We conducted differential gene expression analysis to assess changes within the tumor microenvironment (TME). Validation of key regulatory interactions was performed using qPCR in a cohort of 21 ESCC patients and further substantiated through experimental assays in ESCC cell lines.

**Results:**

The study revealed critical alterations in cell composition and gene expression across identified cell populations, with a notable shift towards pro-tumorigenic states. A significant regulatory influence of STAT3 on LHPP was discovered, establishing a novel aspect of ESCC pathogenesis. Elevated levels of STAT3 and suppressed LHPP expression were validated in clinical samples. Functional assays confirmed that STAT3 directly represses LHPP at the promoter level, and disruption of this interaction by promoter mutations diminished STAT3's repressive effect.

**Conclusion:**

This investigation underscores the central role of STAT3 as a regulator in ESCC, directly impacting LHPP expression and suggesting a regulatory loop crucial for tumor behavior. The insights gained from our comprehensive cellular and molecular analysis offer a deeper understanding of the dynamics within the ESCC microenvironment. These findings pave the way for targeted therapeutic interventions focusing on the STAT3-LHPP axis, providing a strategic approach to improve ESCC management and prognosis.

## Introduction

Esophageal squamous cell carcinoma (ESCC) is a leading cause of cancer death worldwide, particularly in Central and Eastern China where it accounts for about 90% of all esophageal cancer cases. (Siegel et al. 2020; Morgan, et al. 2020; Uhlenhopp et al. 2020) Despite progress in detection and treatment strategies, the outlook for ESCC patients is poor, with late diagnosis and limited treatment options often leading to a five-year survival rate of only 5–25% (Abnet et al. 2018; Thrift 2021). The high incidence of ESCC in this population is thought to be driven by a combination of genetic and environmental factors, and dietary habits (Rustgi and El-Serag 2014; Rumgay et al. 2021).

To tackle the underlying complexities of ESCC, researchers have increasingly relied on comprehensive cancer databases and cutting-edge technologies like single-cell transcriptomics. This innovative approach marks a significant departure from traditional bulk transcriptomics, which averages the data across many cells, obscuring the detailed cellular diversity within tumors. Single-cell RNA sequencing (scRNA-seq) emerges as a powerful, unbiased tool, enabling the classification of cells based on their unique gene expression profiles, independent of any prior genetic or protein markers. This technique provides unparalleled insights into the cellular composition of tumors, highlighting the diversity among cancerous, immune, and stromal cells and revealing potential new strategies for overcoming tumor-induced immune evasion (Regev, et al. 2017; Tanay and Regev 2017; Dominguez Conde, et al. 2022; Yang et al. 2024).

Transcription factors (TFs) stand as the central regulator to the cellular gene expression (Regev, et al. 2017; Wu et al. 2020) TFs such as STAT3 play critical roles in cellular processes ranging from differentiation to proliferation, and in the context of cancer, they are key players in driving tumorigenesis and metastasis (Tolomeo and Cascio 2021; Ma, et al. 2022). Overactivation of STAT3, in particular, results in the heightened expression of genes that contribute to inflammation, proliferation, metastasis, and immunosuppression, collectively facilitating the onset of cancer. (Liu et al. 2013; Zhang et al. 2020) Additionally, STAT3 orchestrates the immune system's response to neoplastic cells, reinforcing the immunosuppressive environment fostered by tumors (Yu et al. 2009). Numerous studies have documented that the activation of STAT3 in cancer cells orchestrates the release of various pro-inflammatory cytokines, including IL-6, IL-10, and VEGF. This activation also impairs the function of natural killer cells, enhancing the ability of tumor cells to evade the immune system while circulating (Hu et al. 2024).Among the genes governed by TFs, LHPP, also known as phospholysine phosphohistidine inorganic pyrophosphate phosphatase. encoding a phosphatase enzyme involved in the dephosphorylation of phospholysine and phosphohistidine residues. LHPP was initially investigated in the context of liver cancer, where it was found to inhibit the growth of autochthonous hepatocellular tumors prompted by the concurrent loss of *PTEN* and *TSCL* genes (Guo et al. 2022; Hindupur et al. 2018). Emerging evidence suggest it plays a vital role in various cellular processes such as cell proliferation, apoptosis, and tumor suppression. Dysregulation of LHPP, particularly its downregulation has been implicated in the pathogenesis of several cancers, associated with tumor progression and poor prognosis (Hou et al. 2021; Zhu et al. 2023). Previous studies have posited LHPP as a potential tumor suppressor, with its downregulation correlating with the exacerbation of oncogenic pathways across various cancers. Exploring the regulatory mechanisms and functions of LHPP is crucial for understanding the pathways involved in ESCC development (Hou et al. 2020; Linder et al. 2023).

This study validated alterations in epithelial, endothelial, and T cell populations within ESCC progression by integrating single-cell analyses, cancer databases and patient cohort data. Our results highlight the significant predictive role of LHPP’s negative regulation by STAT3 in ESCC advancement. By revealing the impact of key regulating genes and pathways in ESCC progression, our study provides a valuable framework for cancer research and potential interventions aimed at combating this challenging cancer.

## Results

### Exploring cellular dynamics in the ESCC microenvironment using single-cell RNA-sequencing.

**Fig. 1:**
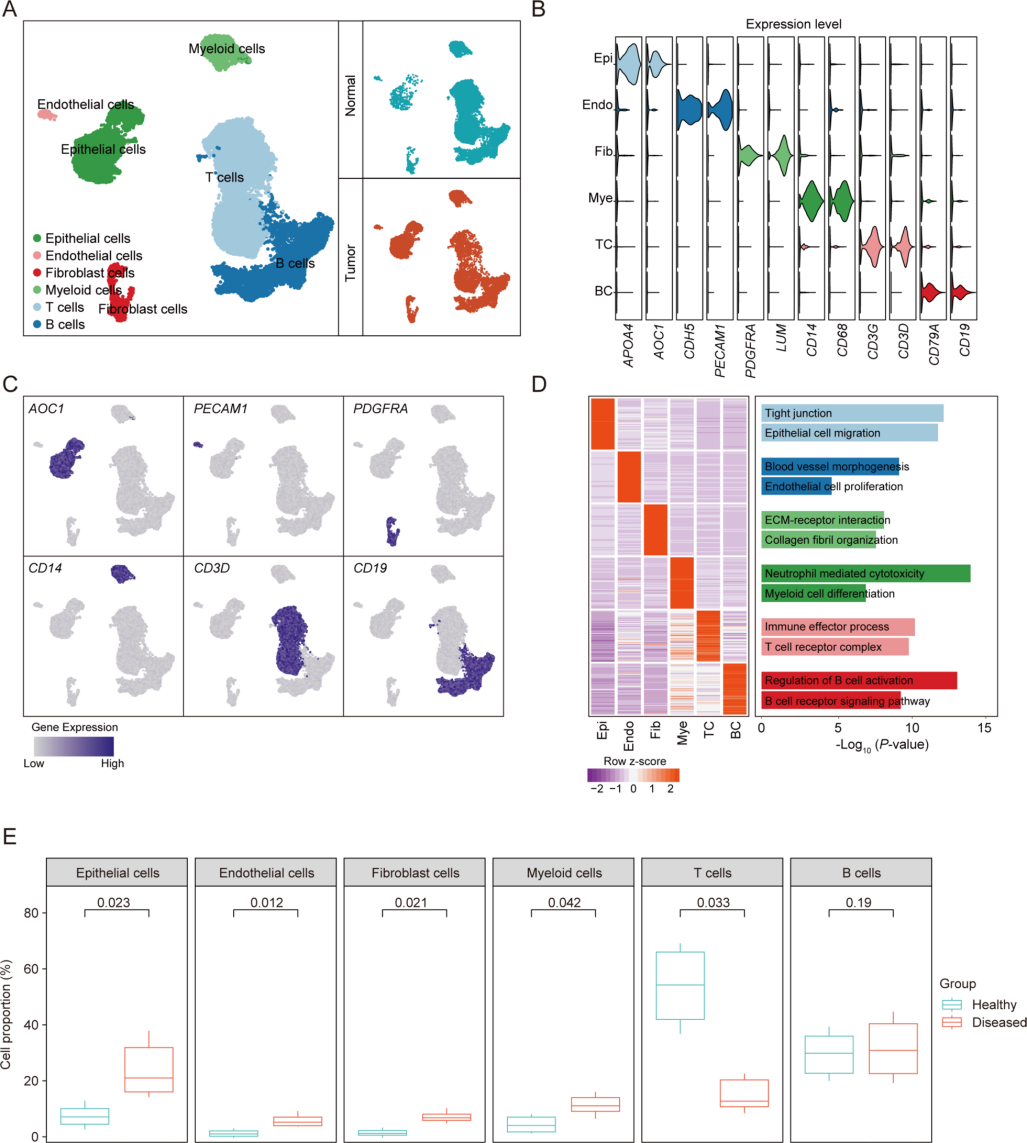
Spectrum of Cell Types Detected through scRNA-Seq Analysis. **A** UMAP plot illustrating the single-cell data distribution across different cell types in ESCC tissues. **B** Violin plot depicting the expression levels of classic marker genes across various cell types. **C** UMAP plots demonstrate the expression patterns of specific marker genes for each cell type. The color gradient from gray to purple indicates increasing levels of gene expression. **D** Heatmap displaying the expression levels of the top 50 marker genes for each cell type, with functional annotations on the right. The color gradient from purple to red denotes lower to higher expression levels. **E** Box plots compare cell type proportions between healthy and tumor tissues, showing differences across epithelial cells, endothelial cells, fibroblast cells, myeloid cells, T cells, and B cells

To unravel the complex changes within the ESCC microenvironment, we utilized single-cell RNA-sequencing data from patient-derived samples, obtained from GSE160269 in GEO database. (Zhang et al. 2021) Detailed analysis of 44,206 cells from both cancerous and surrounding non-cancerous tissues facilitated the identification of six primary cell types: epithelial cells (*AOC1*), endothelial cells (*PECAM1*), fibroblasts (*PDGFRA*), myeloid immune cells (*CD14*), T cells (*CD3D*), and B cells (*CD39*) (Fig. 1A–C). We confirmed these cell populations through the evaluation of known marker genes, confirming the accuracy of our cellular classification (Fig. 1B–D).

Our investigation showed a significant increase in the proportion of Epi (epithelial cells) in the tumor environment (Fig. 1E), suggesting tumor growth and a proliferative index, which mirror the malignancy’s stage and capacity for expansion. Additionally, the rise in Endo (endothelial cells) number indicates an ongoing angiogenic process, essential for tumor sustenance and development. This enhancement of the tumor’s vascular network not only improves the supply of nutrients and oxygen but may also establish pathways for metastatic spread.

The tumor stroma displayed significant fibrotic features, evidenced by a heightened presence of Fib (fibroblasts) (Fig. 1E). This increase suggests a role for these cells in altering the structural and functional aspects of the tumor environment, potentially affecting the cancer's invasiveness and responsiveness to treatment.

A particularly troubling finding was the decrease in T cell proportions within the tumor niche (Fig. 1E), a critical element of the anti-tumor immune response. This decrease hints at an adept evasion of immune surveillance by the tumor, potentially allowing for unchecked tumor progression and underscoring the necessity for a better understanding of the immune landscape in ESCC.

Collectively, the data illuminate the complex cellular interactions within the ESCC microenvironment, emphasizing the roles of proliferative epithelial cells, angiogenic endothelial cells, and fibrogenic fibroblasts, set against the backdrop of a diminished T cell-mediated immune response. These insights not only enhance our comprehension of the biological mechanisms of ESCC but also provide a basis for t creating targeted therapies that focus on these specific cellular activities.

### Exploring transcriptomic changes across cell types during ESCC development

**Fig. 2:**
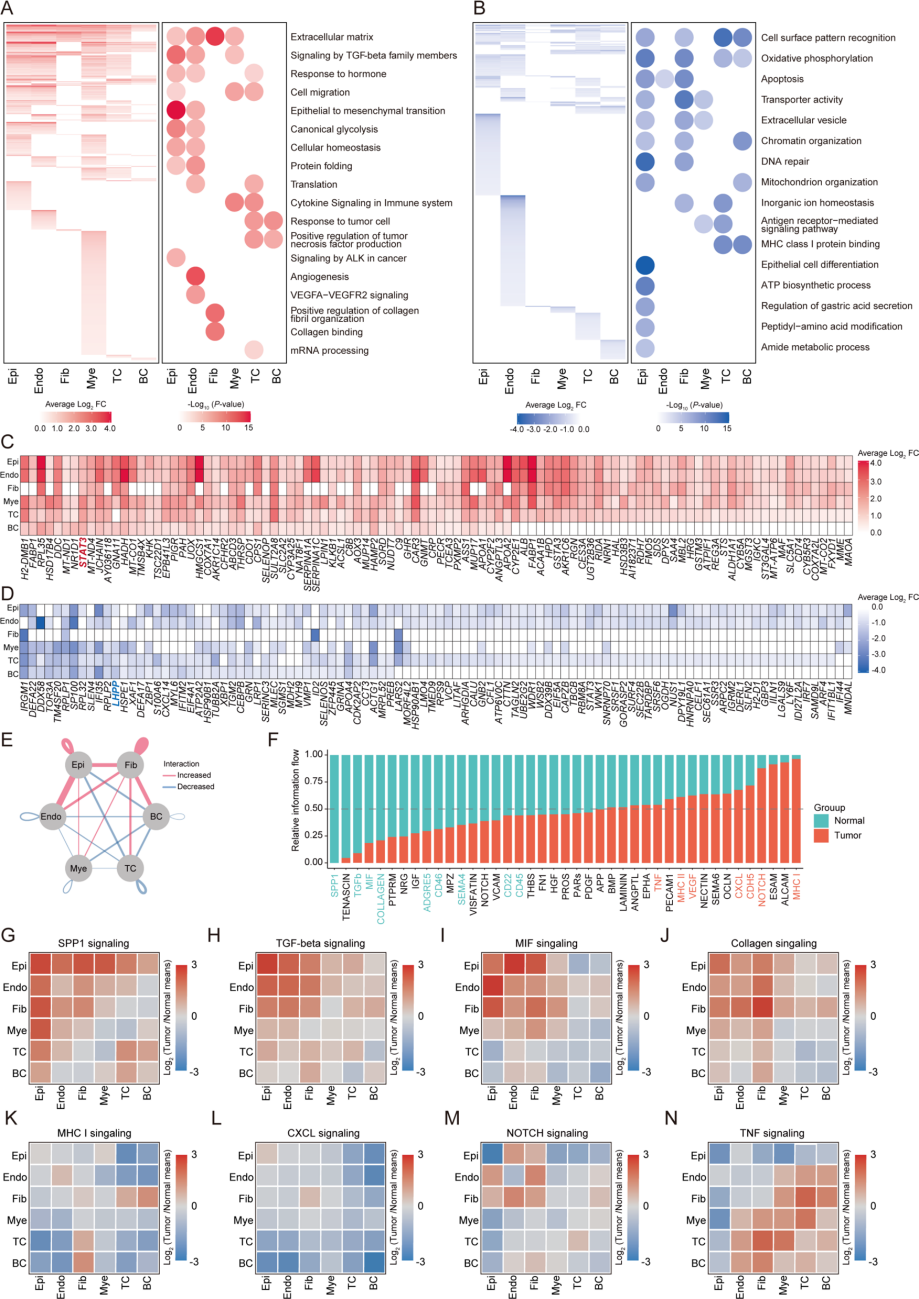
Alterations in the transcriptional landscapes of various cell types throughout tumor genesis. **A** Left: Heatmap displaying genes upregulated in tumor samples relative to healthy controls across various cell types. Right: Dot plot depicting the functional annotations associated with these upregulated genes. **B** Left: Heatmap illustrating genes downregulated in tumor samples compared to healthy controls for each cell type. Right: Dot plot showing the functional annotations of these downregulated genes. **C** Heatmap showing the top 100 upregulated DEGs during tumor genesis. **D** Heatmap showing the top 100 upregulated DEGs during tumor genesis. **E** Interaction network within the ESCC tumor microenvironment, highlighting increased (red lines) and decreased (blue lines) interactions among cell types compared to normal tissue.F: Bar graph showing the interaction flow among signaling pathways in tumor (orange) and normal (green) tissues, with pathways ordered by their proportional intensity in the tumor environment. **G**–**N** Heatmaps depicting the log2-fold change of SPP1 (**G**), TGF-beta (**H**), MIF (**I**), collagen (**J**), MHC I (**K**), CXCL (**L**), NOTCH (**M**), TNF (**N**) signaling across various cell types in tumor tissue relative to normal, with the color gradient shifting from red (indicating upregulation) to blue (indicating downregulation)

Our thorough analysis of gene expression changes across different cell types during the progression of ESCC uncovered extensive transcriptional shifts, with 1482 genes exhibiting increased expression and 1369 showing a decrease. This marked reconfiguration of the genetic framework highlights the cellular adjustments to oncogenic transformation (Fig. 2A–D).

Upon analyzing genes with heightened expression, a distinct pattern emerged underscores the activation of genes associated with fibrotic activities, particularly noted in the enrichment of “Extracellular matrix” and “Signaling by TGF-beta family members”, as well as genes that facilitate “Epithelial to mesenchymal transition (EMT)”. These upregulated pathways indicate a strengthened fibrotic environment within cancer tissues. The increased expression of EMT-related genes highlights crucial morphological alterations in epithelial cells that are essential for cancer development. Furthermore, a significant rise in “Canonical glycolysis” observed in epithelial and endothelial cells points to a metabolic shift that supports cancer progression. Specifically, endothelial cells exhibited a notable increase in angiogenic genes, including “Angiogenesis” and “VEGFA-VEGFR2 signaling”, suggesting active vascular remodeling to accommodate tumor growth. The elevated gene expression in immune cells, notably T cells and B cells, in “Response to tumor cell” and “Positive regulation of tumor necrosis factor production” pathways, indicates a vigorous immune response to cancer cells (Fig. 2A, C).

Conversely, the noted decrease in gene expression linked to “Cell surface pattern recognition” in both epithelial and endothelial cells might indicate strategic immune evasion tactics by the tumor. A metabolic shift was also suggested by the reduction in “Oxidative phosphorylation”, which may promote reliance on glycolytic pathways. Furthermore, the lowered expression of genes essential for “Apoptosis”, “DNA repair”, and “Chromatin organization” hints at potential survival strategies that support malignant cell growth. Additionally, the diminished expression in immune-related pathways, such as “Antigen receptor-mediated signaling pathway” and “MHC class I protein binding”, raises concerns regarding the immune system's capacity to effectively target and eradicate cancer cells (Fig. 2B, D).

The detected gene expression dynamics underscore the transformative condition of the ESCC microenvironment. Increased gene expression in fibroblasts, epithelial, and endothelial cells indicates an adaptive response leading to fibrotic and metabolic modifications. On the other hand, the reduction in specific genes within endothelial, epithelial, and T cells might represent a strategic adjustment by these cells in the tumor setting. Altogether, these variations depict a complex and detailed cellular reaction within the ESCC framework, emphasizing the necessity for a comprehensive understanding of the molecular and cellular mechanisms involved in the advancement of ESCC.

### Comprehensive analysis of cell-cell communication and pathway dynamics in the tumor microenvironment

In our efforts to further clarify the changes in the tumor microenvironment (TME) during tumorigenesis, we explored the detailed cell-cell communication mechanisms among various cell types. We noted a significant increase in the interactions between Epi, Endo, and Fib throughout the tumor development. This escalation highlights the mutual reinforcement between tumor tissues and the adjacent neovasculature, as well as an increased level of fibrosis. Contrarily, immune cells, particularly TC and BC, exhibited a notable decrease in their interactions with other cellular components (Fig. 2E). This reduction further suggests the tumor cells' proficiency in evading immune detection. Overall, our observations emphasize crucial changes in cell-cell communications, indicating a combined enhancement of tumor progression and angiogenesis, alongside a reduced engagement of immune-cell within the TME.

From a broader perspective, our cell-cell communication analysis revealed significant alterations in various pathways within the TME. Key among these were notable upregulations in the SPP1, TGFbeta, MIF, COLLAGEN, and CD45 pathways, contrasted with distinct downregulation in the TNF, MCHI & MHCII, CXCL, CDH5, and NOTCH pathways (Fig. 2F). These modifications shed light on the subtle environmental changes that transpire as the tumor develops.

Exploring cell-specific pathway activities further, we noted significant upregulation of the SPP1 pathway, particularly in Epi, Endo, Fib, and Mye (myeloid immune cells). Its widespread elevation across these cell types highlights its essential role in facilitating diverse interactions within the TME (Fig. 2G). Both the TGF-beta and MIF pathways exhibited notable upregulation in Epi, Endo and Fib cells, indicating their combined impact on cellular growth, matrix alteration, and immune evasion within the tumor environment (Fig. 2H, I). Specifically, The COLLAGEN pathway was prominent in Fib cells as they engaged with other cells, emphasizing the fibroblasts’ primary role in adjusting the extracellular matrix, which may affect tumor stiffness and migration (Fig. 2J).

Conversely, the MHC and CXCL pathways showed a significant decrease in activity, particularly in the interactions between TC and BC with other cellular entities (Fig. 2K, L), potentially aiding immune evasion and giving tumor cells a hidden advantage in the TME. This noticeable reduction may be a strategic modification by tumor cells to avoid detection and suppression by the immune system. The NOTCH pathway displayed dual behavior, with upregulation in EC enhancing angiogenesis, thus encouraging the formation of new blood vessels. However, its downregulation in Epi cells suggests a change in epithelial tissue connectivity, marking a transformation towards tumorigenic traits (Fig. 2M). The TNF pathway, crucial for inflammatory responses, was upregulated in interactions among immune cells, reflecting the body's intensified response to the tumor, indicative of efforts to inhibit its growth. Nevertheless, its downregulation in Epi and Endo cells may signify the tumo’s advancement, reflecting the adaptive strategies the tumor employs to ensure its survival and progression (Fig. 2N).

In summary, our research offers a detailed view of the dynamic interactions among various signaling pathways and their cell-specific behaviors. The varied regulations across these pathways not only underscoret the tumor's adaptive strategies but also illuminate the changing communications within the TME, potentially providing insights for therapeutic interventions.

### Tracing epithelial cell transformations and gene expression patterns in ESCC development

**Fig. 3:**
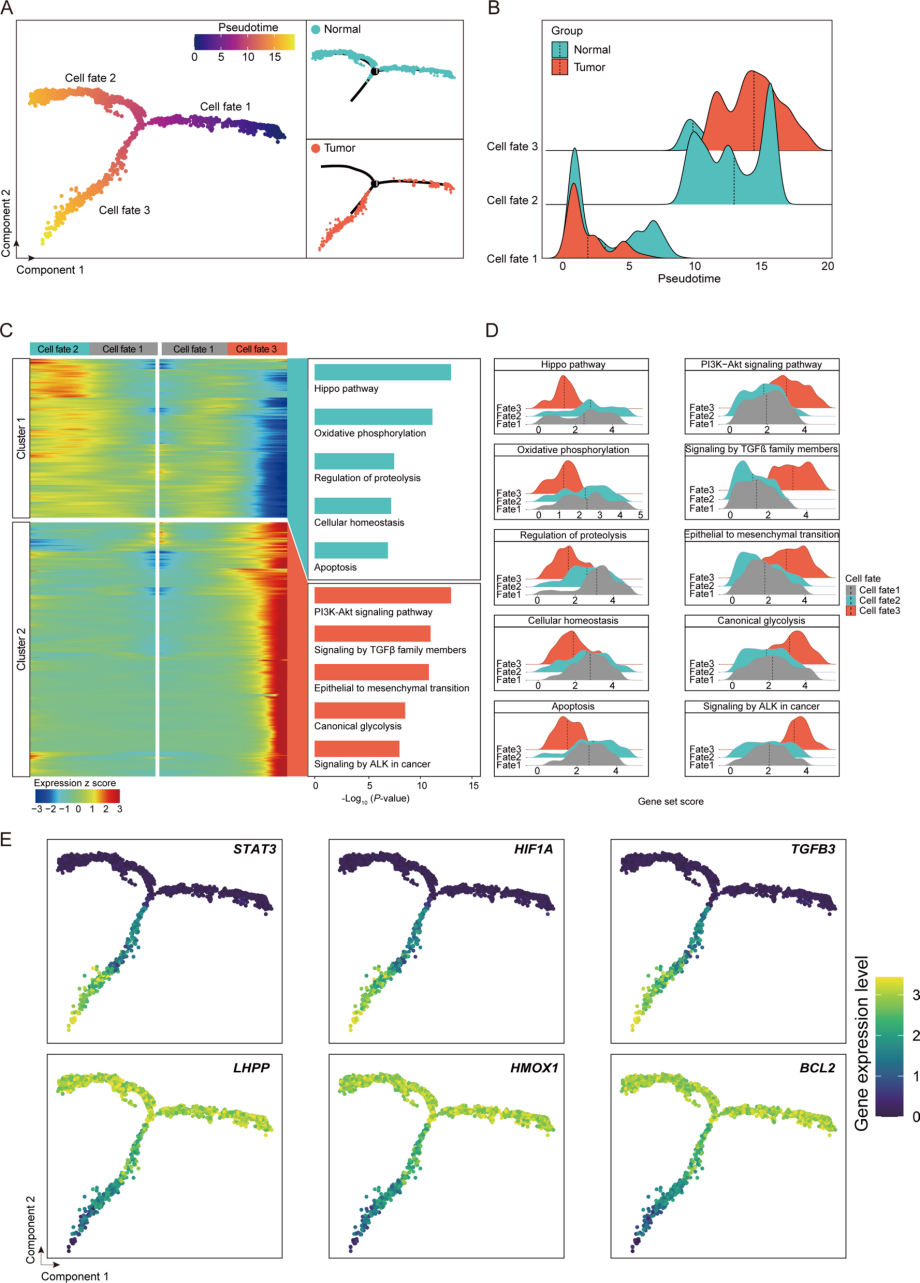
The cellular and molecular transformations of epithelial cells across tumor progression. **A** Pseudotime trajectory plot of of epithelial cells in ESCC. Left, pseudotime sequence scores of epithelial cells. Top right, the distribution of epithelial cells in healthy group. Bottom right, the distribution of epithelial cells in tumor group. **B** Ridge plot showing the cell proportion distribution of healthy and tumor epithelial cells along pseudotime trajectory of **A**. **C** Heatmap showing the time-related gene expression profiles during tumor genesis, with gene function annotation on the right. **D** Ridge plots showing the expression score of gene set from different clusters in **C** of healthy and tumor groups. **E** Scatter plots and trajectory plots illustrating the expression levels of key genes identified in **C**

In our detailed exploration of the specific changes that epithelial cells experience during ESCC development, we applied pseudotime analysis to map out the different cellular states across the carcinogenic progression of these cells. Notably, our analysis delineated three unique cellular states, each shedding light on various behaviors of epithelial cells throughout ESCC progression: "Cell state 1" was present in both normal and ESCC tissues, likely indicative of typical epithelial cells. "Cell state 2," primarily found in normal tissues, seemed to represent the normal differentiation pathway of epithelial cells. Conversely, "Cell state 3," mainly observed in ESCC tissues, appeared to mark the onset of malignant transformation in epithelial cells (Fig. 3A, B). The progression from "cell state 1" to "cell state 2" reflects standard physiological differentiation, whereas the transition to "cell state 3" suggests the pathological shift from normal to cancerous states, underscoring the intricate evolution of epithelial cells from normality to malignancy in ESCC.

In our efforts to decode the dynamic gene expression shifts during ESCC development, we analyzed gene expression variations along two distinct differentiation trajectories, identifying two pivotal gene clusters with specific roles in the transformation of epithelial cells in ESCC (Fig. 3C, D).

Cluster 1 comprises genes that show a decrease in expression within the ESCC environment while either maintaining or increasing expression during normal differentiation. This cluster contains genes critical for for ESCC progression, including those linked to the Hippo pathway, whose downregulation suggests an ESCC-induced subversion of growth control and tissue structure. Additionally, genes involved in Oxidative phosphorylation are reduced, indicative of a metabolic shift towards glycolysis, essential for cancer cell proliferation in ESCC. The regulation of proteolysis gene group indicates a potential ESCC-induced disturbance in proteostasis, possibly enhancing metastatic potential. A reduction in genes essential for cellular homeostasis marks a disruption in internal balance, crucial in ESCC development. Furthermore, the reduction of apoptosis-associated genes points to an ESCC- promoted avoidance of cell death, contributing to cellular immortality.

Conversely, Cluster 2 encompasses genes that are upregulated during ESCC development, featuring genes crucial to oncogenesis. This includes the PI3K-Akt signaling pathway, a key conduit for cell survival and proliferation, whose upregulation suggests a heightened potential for ESCC progression. The increase in genes associated with signaling by TGFβ family members highlights enhanced interactions with TGF-β, a major regulator of cell fate and oncogenesis. An increase in genes facilitating the epithelial to mesenchymal transition illustrates the vital phenotypic shift that equips epithelial cells with invasive and migratory traits, fundamental to ESCC metastasis. The heightened expression of canonical glycolysis genes reflects a metabolic shift towards a glycolytic phenotype, a strategic adaptation of ESCC cells. Additionally, the rise in genes related to signaling by ALK in cancer highlights a possible oncogenic signaling hub within ESCC.

In the complex landscape of ESCC, our focused gene expression analysis has pinpointed crucial genes that mark the transition from normalcy to malignancy (Fig. 3E). The genes *STAT3*, *HIF1A*, and *TGFB3* emerge as upregulated markers in the tumorigenic process, maintaining stability in non-malignant tissues. *STAT3*, with its varied roles in transcriptional regulation, seems integral to ESCC progression, potentially guiding cellular proliferation and immune evasion. The notable rise in HIF1A expression, key in regulating responses to low oxygen levels, highlights its likely role in neovascularization and the aggressive behavior of tumor cells in the hypoxic areas of ESCC. Furthermore, *TGFB3*'s involvement in the TGF-β signaling pathway suggests its role in altering immune responses and fostering oncogenic growth. In stark contrast, the genes *LHPP*, *HMOX1*, and *BCL2* are markedly downregulated in the ESCC setting, indicating a shift in their regulatory functions within tumors. The decreased expression of LHPP suggests a reduction in its tumor-suppressive effects, potentially facilitating ESCC pathogenesis. The diminished expression of HMOX1 could indicate a compromise in antioxidative defenses, possibly heightening oxidative stress—a condition that could be leveraged by proliferating ESCC cells. Moreover, the lowered expression of *BCL2*, typically involved in inhibiting cell death, hints at a subtle susceptibility of ESCC cells to apoptotic triggers, an aspect critical to understanding the survival strategies of ESCC cells.

In conclusion, these dynamic gene changes gene expression provide deep insights into the cellular transformations occurring during ESCC development. This s research sheds light on the intricate molecular drivers of these processes and opens avenues for further investigation and potential therapeutic interventions in ESCC.

### STAT3 was pinpointed as a central regulator in the advancement of ESCC

**Fig. 4:**
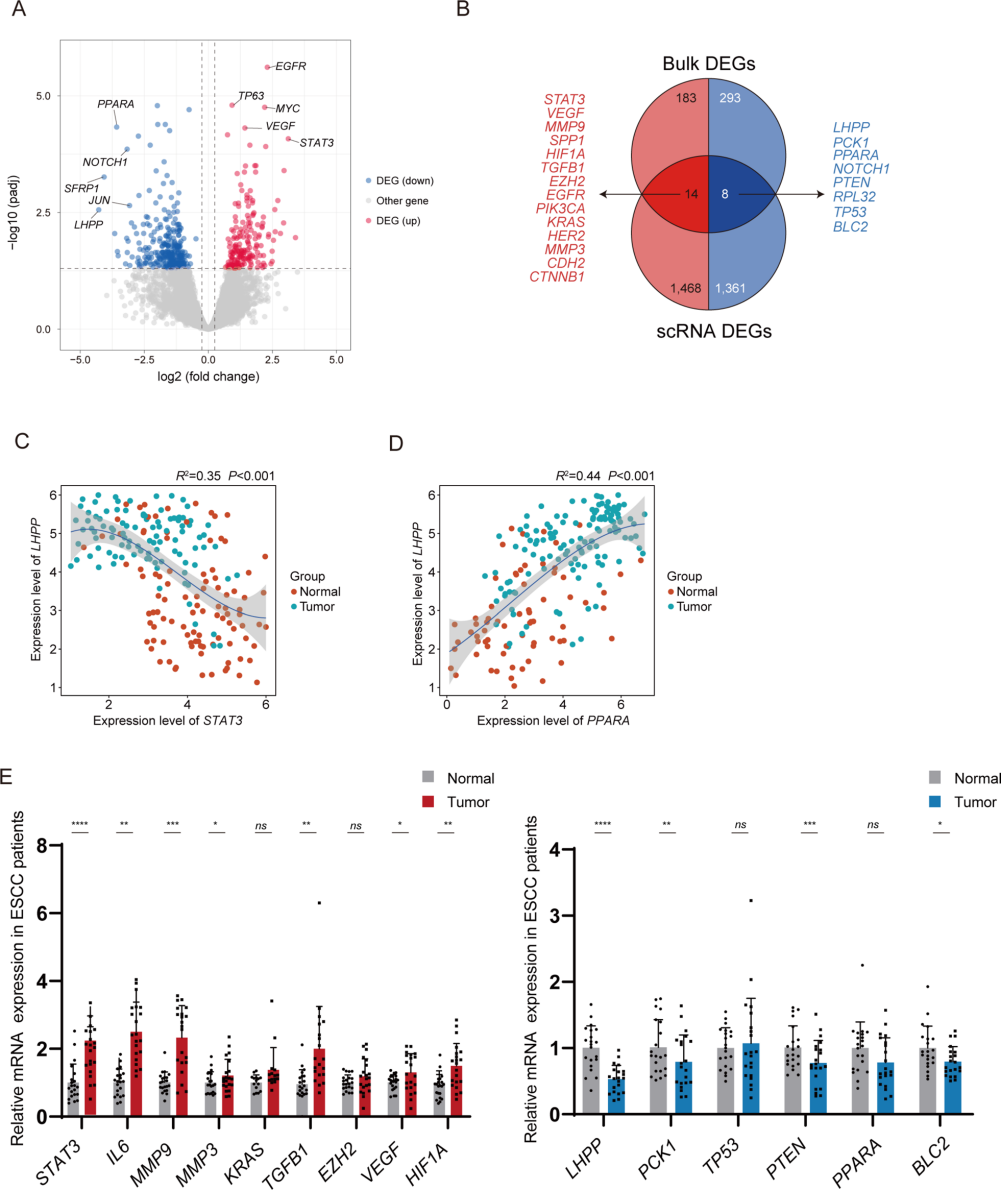
STAT3 was identified as a central regulator in ESCC progression. **A** Volcano plot showing the DEGs distribution in bulk RNA-sequencing data. **B** Venn plot showing the overlap between scRNA-seq DEGs and bulk RNA-seq data. **C** Scatter plot displays the correlation between *LHPP* and *STAT3* expression across normal (orange dots) and ESCC (green dots) tissue samples. **D** Scatter plot displays the correlation between *LHPP* and *PPARA* expression across normal (orange dots) and ESCC (green dots) tissue samples. **E** The relative mRNA levels of the overlap DEGs were detected in 21 paired ESCC patient tissues

To further explore the regulatory mechanisms underlying ESCC pathogenesis, we utilized a list of differentially expressed genes (DEGs) list derived from the ESCC disease database (https://tcga-xena-hub.s3.us-east-1.amazonaws.com/download/TCGA.ESCA.sampleMap%2FHiSeq.gz) and performed a comprehensive integrative analysis with our scRNA-seq dataset (Fig. 4A, B). Our analysis identified 14 consistently upregulated genes (*STAT3, VEGF, MMP9, SPP1, HIF1A, TGFB1, EZH2, EGFR, PIK3CA, KRAS, HER2, MMP3, CDH2, CTNNB1*) and 8 genes that were downregulated. Notably, the significant downregulation of *LHPP*, which encodes a phosphatase involved in the dephosphorylation of phospholysine and phosphohistidine residues, stood out. Previous research has linked its downregulation with tumor advancement and adverse outcomes. (Guo et al. 2022; Hindupur et al. 2018; Zhu et al. 2023) Further correlation analysis between *LHPP* and transcription factors of the differentially expressed genes showed a notable negative association with *STAT3* and a positive correlation with *PPARA* (Fig. 4C, D).

To validate our findings, we recruited a cohort of 21 cancer patients for histological confirmation, revealing significant mRNA upregulation of *STAT3*, *IL6*, *MMP9*, *MMP3, TGFB1*, *VEGF* and *HIF1A*, coupled with downregulation of *LHPP* and *PCK1, PTEN* and *BLC2* (Fig. 4E). However, alterations in *EZH2* and *PPARA* expression were not statistically significant. These findings suggest a potential transcriptional inhibitory role of STAT3 on LHPP expression.

### STAT3 plays a negative regulatory role in LHPP expression in ESCC

**Fig. 5:**
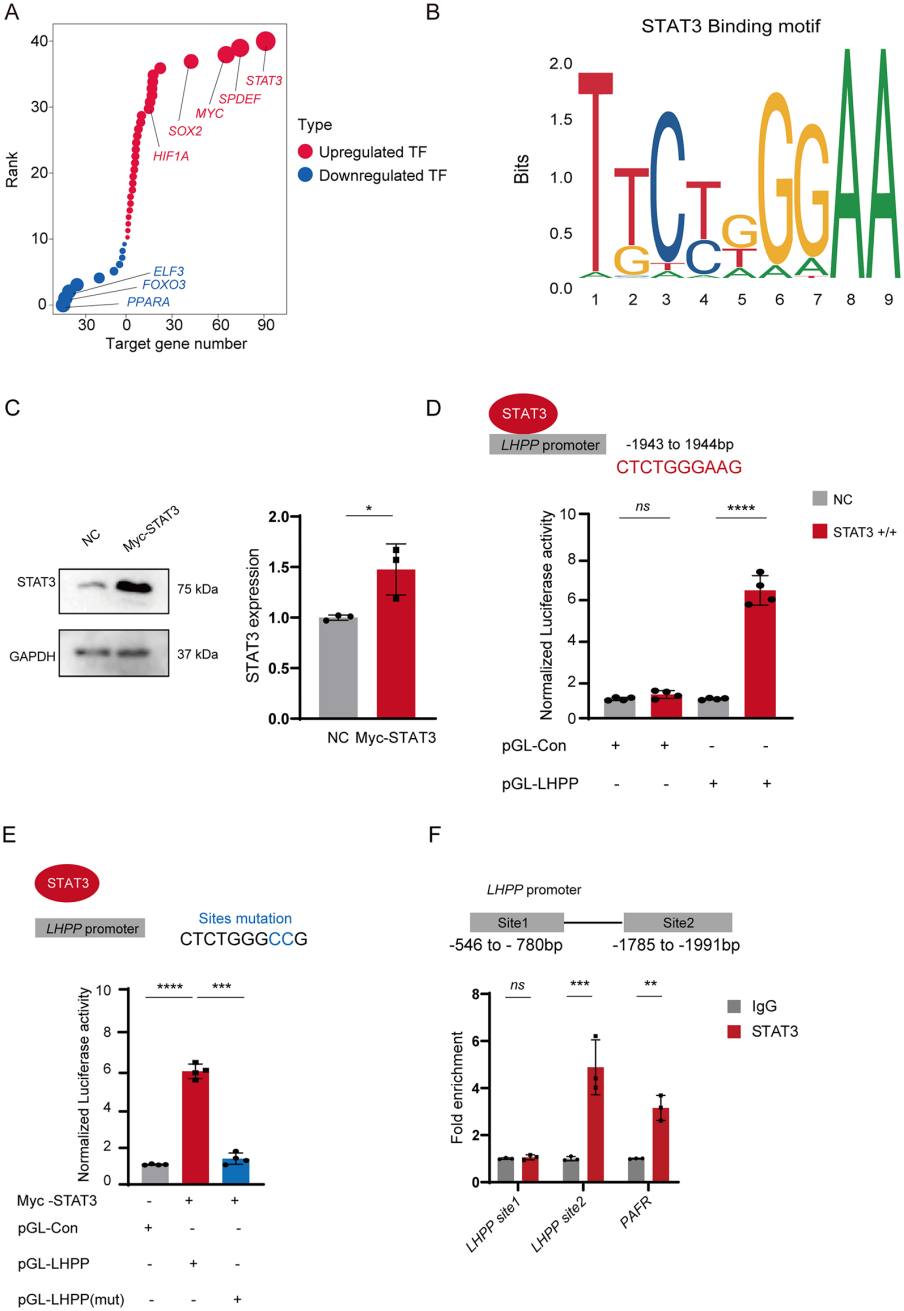
STAT3 Plays a Negative Regulatory Role in LHPP Expression in ESCC cells. **A** Dot plot showing the upregulated (red) and downregulated TFs, with size indicating the target gene number. **B** Representation predicted binding motif of STAT3. **C** Western blot analysis displaying the overexpression levels of STAT3 proteins in EC9706. **D** Luciferase reporter assay results showing the effect of STAT3 on the LHPP promoter activity in EC9706. **E** Luciferase reporter assays illustrating the lack of suppressive activity in promoter Mutation compared to the wild-type promoter in the presence of STAT3. **F** ChIP-qPCR analysis showing STAT3 binding to the *PAFR* and *LHPP* promoter in KYSE-150 cells

To delve deeper into the regulatory roles of *STAT3* in *ESCC*, we undertook transcription factor prediction using these differentially expressed genes. Our findings highlighted *STAT3*, *SOX2*, *FOXO3*, and *PPARA* as the most significant transcription factors, possess the largest number of target genes, underscoring their critical roles in gene regulation within ESCC (Fig. 5A). Coupled with the prediction analysis of the STAT3 transcription factor binding motif, we identified a binding site for STAT3 in the promoter region of *LHPP* (Fig. 5B).

In the ESCC cell line EC9706, we employed Luciferase reporter assays to validate the binding of STAT3 to the *LHPP* promoter region. Our result confirmed the activation of the LHPP promoter in the presence of overexpressed STAT3, indicating a functional binding of STAT3 to this specific promoter region (Fig. 5C, D). These findings in the progression of ESCC disease indicate that STAT3 negatively regulates LHPP expression by binding to its promoter region, suggesting a significant transcriptional inhibitory role that contributes to the pathophysiology of the disease. To further dissect the mechanism underlying STAT3's influence on LHPP promoter activity, we introduced a point mutation at the putative STAT3 binding site within the promoter region (Fig. 5E). This mutation altered the consensus sequence, hypothesized to be critical for STAT3's interaction. The luciferase activity measurements post-mutation, shown in Fig. 5E, revealed a significant reduction to near-baseline levels, underscoring the essential role of this binding site for STAT3-mediated transcriptional regulation. Based on predictions from binding motifs and validations through luciferase reporter assays and site-directed mutagenesis, we identified a potential positive STAT3 binding site at Site2 (− 1786 to − 1991 bp) and a potential negative site at Site1 (− 546 to − 780 bp) in the promoter region of *LHPP*. Additionally, we used previously published ChIP-on-chip data from three ESCC cell lines, which revealed STAT3 binding sites on the *PAFR* gene, serving as an additional experimental control (Zhao et al. 2023). ChIP-qPCR results in KYSE-150 cells confirmed the enrichment of STAT3 at the *LHPP* promoter's Site2 and on the *PAFR* gene, aligning with our findings from luciferase assays. Notably, no enrichment was observed at the negative control site1, further validating the specificity of STAT3 binding in our study. This consistency between ChIP-qPCR and luciferase data reinforces the reliability of our conclusions regarding the regulatory interactions of STAT3.

Collectively, our findings highlight the profound capability of single-cell genomics to demystify the complex molecular landscape of ESCC. The identification of pivotal transcription factors such as *STAT3*, and their associated expression patterns, not only deepens our comprehension of the disease's pathophysiology but also directs us towards more precise and potentially effective therapeutic interventions. Importantly, the discernment of STAT3's regulatory role suggests its critical influence in the progression of ESCC, offering a promising target for future treatment strategies.

## Discussion

Single-cell sequencing illuminated the intricate gene regulatory networks and detailed cellular interactions within cancer research, significantly enhancing our understanding of the complex tumor microenvironment (Lei et al. 2021). In this study, we validated the changes in epithelial, endothelial, and T cell populations during the progression of ESCC through single-cell database. Our single-cell transcriptomic analysis has revealed major cellular shifts within the ESCC progression. The increase in epithelial cells h underscores the carcinoma's aggressiveness, given their role as primary contributors to tumor mass. A concomitant surge in endothelial cells is indicative of angiogenesis, essential for tumor nourishment and metastatic dissemination. Additionally, an increase in fibroblasts suggest an enhanced fibrotic reaction, which is often associated with tumor rigidity and potentially influence the invasive and metastatic potential of the cancer. These observations affirm that the ESCC microenvironment is not simply passive but actively involved in facilitating disease progression. Moreover, a notable reduction in T cells within the tumor milieu is particularly concerning. As the sentinels of the immune system, T cells play a crucial role in recognizing and destroying cancer cells (Zhao et al. 2021). Their diminished presence likely reflects an immune evasion tactic employed by the tumor, posing a significant challenge to immunotherapeutic strategies.

Analysis of cell-type-specific transcriptomic alterations during ESCC progression reveals a marked upregulation of genes involved in processes such as fibrosis, EMT, and glycolysis. This gene expression profile correlates with the observed cellular composition changes, where the tumor environment is becoming more conducive to cancer growth and survival. Conversely, the downregulation of genes involved in immune recognition, apoptosis, and DNA repair within the tumor suggests mechanisms that may contribute to the cancer's resilience and persistence (Whiteside 2008).

The differential expression patterns of the genes *STAT3*, *HIF1A*, *LHPP*, *HMOX1*, and *BCL2* underscore the complexity of the tumorigenic process. The upregulation of *STAT3*, a transcription factor known for its role in cell survival and immune evasion, along with *HIF1A* facilitates a cellular environment conducive to tumor growth and immune system suppression. Conversely, the downregulation of *LHPP*, *HMOX1*, and *BCL2* disrupt tumor-suppressive functions, oxidative stress management, and apoptotic processes, respectively.

The role of STAT3 is further emphasized by its identification as a core regulator in ESCC. Our transcription factor analysis and subsequent validation by qPCR analysis confirm the significant upregulation of *STAT3* in ESCC patient tissues. Our analysis involving transcription factor binding sites, coupled with luciferase assay validations, illustrates that STAT3 exerts a negative regulatory influence on LHPP by binding to its promoter region. This interaction is further corroborated by ChIP-qPCR, which confirms the presence of STAT3 at this locus. Earlier research has connected LHPP with crucial pathways involving mTOR and the DNA replication factor P53, revealing complex molecular dynamics (Hindupur et al. 2018). Notably, STAT3 acts as a direct target for mTOR, which induces phosphorylation at STAT3's S727 site, and it can also directly engage with the P53 promoter. This dual interaction, particularly in the context of disrupted wild-type P53 and accumulation of mutant P53, facilitates STAT3-driven support for tumor cell proliferation and survival (Dodd et al. 2015; Schulz-Heddergott, et al. 2018). These findings suggest a significant linkage between STAT3 and LHPP, underscored by the elevated expression of STAT3 observed in ESCC tissues and cell lines. Furthermore, STAT3's profound impact on the cellular inflammatory environment, highlighted by increased IL6 levels, may directly foster alterations in the tumor microenvironment, thereby affecting the phosphorylation and functional dynamics of LHPP (Huang et al. 2022).

Our findings collectively enhance our understanding of the cellular and molecular intricacies of ESCC. By detailing the cell-type-specific changes and gene expression profiles, we provide critical insights into the disease's biology and identify potential therapeutic targets. For instance, approaches aimed at reversing the immunosuppressive TME, addressing the metabolic adaptations in cancer cells, or inhibit the pro-tumorigenic effects regulated by STAT3.

In summary, our comprehensive analysis offers an in-depth view of the ESCC landscape, spanning the cellular makeup of the TME to the complex gene expression shifts that propel the disease. By elucidating the roles of essential genes and pathways in the progression of ESCC, our research lays a solid foundation for future investigations and potential treatments aimed at tackling this formidable cancer.

## Limitations

While this study provides significant insights into the interactions between STAT3 and LHPP, it presents several limitations that need addressing in future research. Primarily, the interactions detailed here require further substantiation through protein-level investigations in patient tissues, the use of comprehensive pathological techniques such as immunohistochemistry and immunofluorescence across whole tissue landscapes would provide a more detailed and nuanced view of disease biomarkers and their progression, which could solidify the connection between STAT3 and LHPP in the context of disease mechanisms. Additionally, examining inflammatory markers in plasma could deepen our understanding of the functional implications of these interactions in a systemic context.

Furthermore, extending studies to include ESCC tumor cells from a diverse genetic background would also enhance the validation of transcriptional regulation and its significance. Employing advanced genomic techniques such as RNA-seq and ChIP-seq to map the downstream regulatory networks could enrich our understanding of STAT3 mechanisms. Employing mouse tumor models could serve as a next step to verify the effectiveness of therapeutic interventions Future research, especially those utilizing multispecies disease models, is poised to substantially enhance our comprehension of STAT3's pivotal role in ESCC. Such studies could lead to groundbreaking insights into the disease's mechanisms and novel therapeutic strategies.

## Materials and methods

### Patients and tissues

Biopsy samples from patients clinically diagnosed with ESCC, encompassing tumor and adjacent tissues were collected in the Department of Radiation Oncology, Huaian Hospital of Huaian City (Huaian, China) through 2023. The clinical protocol was approved by the Institutional Ethics Committee of Huaian Hospital of Huaian City (No.2022005) and was in concordance with the Helsinki Declaration. All patients were adult residents of China, and samples were taken without any treatment interference. Collected specimens were immediately preserved in saline chilled on ice and subsequently placed in liquid nitrogen for future analysis.

### Construction of cell atlas of ESCC patients

Gene expression data for healthy and tumor-derived ESCC tissues were obtained from the Gene Expression Omnibus (GEO) under accession GSE160269 (Zhang et al. 2021).GSE160269 Analysis was conducted using the Seurat R package (version 4.0.2). Initial data processing involved the creation of Seurat objects for each ESCC sample using the ‘CreateSeuratObject’ function. Gene expression normalization, scaling, and identification of highly variable genes were performed using ‘SCTransform’. Data integration was accomplished by first selecting integration anchors with ‘PrepSCTIntegration’ and ‘FindIntegrationAnchors’, followed by merging the data using ‘IntegrateData’. Subsequent scaling of the integrated dataset was performed with ‘ScaleData’. For dimensionality reduction, principal component analysis and uniform manifold approximation and projection (UMAP) were executed using ‘RunPCA’ and ‘RunUMAP’, respectively. Cell clustering and identification were achieved using ‘FindNeighbors’ and ‘FindClusters’. Lastly, marker genes for each ESCC cell type were identified using ‘FindAllMarkers’ with thresholds set for average log2 fold change (|avg_log2FC| ≥ 0.5) and adjusted p-value (p_val_adj ≤ 0.05).

### Identification of DEGs between healthy and tumor group across tissues and cell compartments

Differential expression analysis between tumor and healthy groups for each cell type was performed using the ‘FindMarkers’ function in Seurat. This analysis was based on normalized data and employed the Wilcoxon test. The screening criteria for significantly differentially expressed genes were selected by BH-adjusted *P* value ≤ 0.05 and |log_2_FC| ≥ 0.25.

### Cell-cell interaction analysis in ESCC patients using CellPhoneDB

CellPhoneDB were utilized for elucidating intercellular communication networks within the analyzed cell populations. Initially, we prepared a normalized scRNA-seq dataset, ensuring it included annotations for cell types or clusters. We utilized the ‘cellphonedb method’ function to conduct the analysis, which involved the statistical assessment of potential ligand-receptor interactions between the defined cell types. This function compares the input data against a curated database of known ligand-receptor pairs, calculating the probability of their interactions based on expression levels. We selected cell interactions with a p-value ≤ 0.05 to refine our analysis. The output generated by CellPhoneDB, which includes a list of statistically significant interacting pairs along with their corresponding p-values and cell types, was further explored to identify key communication pathways pertinent to our study.

### Pseudo-time trajectory inference

To characterize the mobilization and of ESCC during tumor genesis, the R package Monocle2 was used to perform pseudotime trajectory inference for EC of healthy and tumor tissues. The top 3000 high variable genes were used to calculate the pseudotime. The functions “plot_pseudotime_heatmap” and “plot_genes_in_pseudotime” were used to perform time-related gene analysis.

### Gene set score analysis

The gene sets were downloaded from MSigDB (https://www.gsea-msigdb.org/gsea) and the ‘AddModuleScore’ function of Seurat was used to calculate gene set scores.

## Differential expression analysis in RNA-seq

To identify genes with significant changes in expression between ESCC and adjacent normal tissue samples, we conducted differential expression analysis. This process entails applying a statistical model to the normalized count data. Specifically, we employed the negative binomial model via the DESeq2 software, which is adept at handling count-based expression data. During this analysis, genes were categorized as differentially expressed genes (DEGs) if they met specific criteria: an absolute log2 fold change greater than 0.25 and an adjusted p-value less than 0.05. These thresholds ensure that only genes with statistically significant and biologically relevant changes in expression are considered as DEGs.

### Cell culture and plasmids

The human esophageal squamous carcinoma cell lines EC9706 and KYSE-150 were provided by the Chinese Academy of Sciences Cell Bank Type Culture Collection (Shanghai, China). The Myc-STAT3 overexpression plasmid (NM_213662) was purchased by youbio company (shanghai, China). The LHPP promoter was cloned from the total DNA of EC9706 and constructed into the pGL3 expression vector using a rapid DNA ligation kit (beyotime, D7001M)

### Quantitative real-time PCR

Samples and cells were subjected to physical homogenization and total RNA extracted by TRIzol Reagent (cat. 15596026, Invitrogen) according to the previous reported manufacturer’s instructions (Chen, et al. 2024). For cDNA synthesis, 5ug RNA was reverse transcribed with the high-capacity reverse transcription kit (cat. 4368814, Thermo Fisher). The qPCR reactions were performed using TaqMan gene expression and assays on ABI QuantStudio 5 (Applied Biosystems, Thermo-Fisher).

### Western blotting

Cells were lysed using SDS lysis buffer (P0013G, Beyotime, supplemented with 1 x protease inhibitors (cat. 04693132001, Roche)). Protein was quantified using the BCA Kit (PA115, Tiangen). 20ug proteins were subjected to SDS-PAGE electrophoresisAnd transferred to the PVDF membranes (Millipore), following blocked with 5% skim milk and incubated overnight at 4 ℃ with the following antibodies: mouse monoclonal antibody against GAPDH (1:2500 dilution; Santa Cruz Biotechnology; sc365062); Rabbit monoclonal antibody against STAT3 (1: 1500 dilutions; abcam; ab68153). Following incubation with HRP-conjugated secondary antibodies, the membrane was visualized using an enhanced chemiluminescence kit (cat34580, Thermo) and quantified with ImageJ software.

### Chromatin immuno-precipitation (ChIP)-qPCR

KYSE-150 cells were cultured until they reached 80% confluency. Approximately 1 x 10 (Rustgi and El-Serag 2014) cells were subjected to sonication using a Bioruptor sonicator (Diagenode) to generate chromatin fragments ranging from 200 to 500 bp. The fragmented chromatin was then incubated with 20 µg of a STAT3-specific antibody (abcam; ab68153). Following this, the chromatin-antibody complex was crosslinked to magnetic beads (no. 112.04, Dynal Biotech, Invitrogen). After the crosslinking step, the crosslinks were reversed by heating the samples at 65°C for 16 hours. The precipitated DNA was subsequently treated with Proteinase K and RNase A and purified using the QIAquick PCR Purification Kit (QIAGEN).

For the ChIP-qPCR, primers were specifically designed to amplify STAT3-binding regions (Zhao et al. 2023). The qPCR reactions were performed using TaqMan gene expression and assays on ABI QuantStudio 5 (Applied Biosystems, Thermo-Fisher).

### Statistical analysis

Data are statistically analyzed using the GraphPad Prism 8.0 software. Results are presented as the mean ± SEM. Statistical analyses were performed using a two-tailed Student’s t-test to compare the differences between different groups. *P* values < 0.05 was considered statistically significant (*).

## Data Availability

The datasets used and/or analyzed in the current study are available from the corresponding author upon reasonable request. The raw data of healthy and ESCC human tissues were from GSE160269 in GEO database.
